# A Multiplex PCR for Simultaneous Detection of Three Zoonotic Parasites *Ancylostoma ceylanicum*, *A. caninum,* and *Giardia lamblia* Assemblage A

**DOI:** 10.1155/2015/406168

**Published:** 2015-08-31

**Authors:** Wei Hu, Sheng Wu, Xingang Yu, Auwalu Yusuf Abullahi, Meiran Song, Liping Tan, Zhen Wang, Biao Jiang, Guoqing Li

**Affiliations:** Guangdong Provincial Zoonosis Prevention and Control Key Laboratory, College of Veterinary Medicine, South China Agricultural University, Guangzhou, Guangdong 510642, China

## Abstract

*Ancylostoma ceylanicum*, *A. caninum*, and *Giardia lamblia* assemblage A are common intestinal parasites of dogs and cats; they can also infect humans, causing parasitic zoonoses. In this study, a multiplex PCR method was developed for simultaneous identification and detection of those three zoonotic parasites. Three pairs of specific primers were designed based on ITS sequence of *A. ceylanicum* and *A. caninum* and TPI gene of *G. lamblia* available in the GenBank. The multiplex PCR reaction system was established by optimizing the reaction condition, and a series of tests on the sensitivity, specificity, and clinical application were also conducted. Results showed that three target fragments were amplified specifically; the detection limit was 10 eggs for both *A. ceylanicum* and *A. caninum*, 72 pg DNA for *G. lamblia*. Of 112 clinical fecal samples, 34.8% and 17.8% samples were positive for *A. caninum* and *A. ceylanicum*, respectively, while only 2.7% samples were positive for *G. lamblia* assemblage A. It is concluded that the established multiplex PCR assay is a convenient, rapid, cost-effective, and high-efficiency method for molecular detection and epidemiological investigation of three zoonotic parasites.

## 1. Introduction

Hookworms are blood-feeding parasitic intestinal nematodes which infect humans, dogs, cats, and other mammals throughout the world. The common canine and feline hookworms are* A. ceylanicum*,* A. caninum*,* A. braziliense*,* A. tubaeforme*, and* Uncinaria stenocephala *[1]. Of those species, only* A. ceylanicum *can readily develop to adults in the intestine of humans, resulting in iron-deficiency anemia [2]. Recently, hookworm infection in human caused by* A. ceylanicum *has been reported in Thailand, Laos, Malaysia, and Cambodia [3–7]. The larvae L3 of* A. caninum* are important causative agents not only of eosinophilic enteritis [8], but also of cutaneous larva migrans in humans. Other manifestations in humans include unilateral subacute neuroretinitis, eosinophilic pneumonitis, localized myositis, folliculitis, erythema multiforme, or ophthalmological manifestations [1].

*G. lamblia* is also a serious zoonotic parasite that infects many mammals, including dogs and cats. Infection in human can lead to abdominal cramps, acute or chronic diarrhea, and malabsorption [9]. To date, among the eight assemblages of* G. lamblia* found, only assemblages A and B are associated with human infections but are also recovered from a broad range of hosts, including dogs and cats [10]. Recent epidemiological surveys in China show that the* G. lamblia *zoonotic assemblage infected dogs and cats was assemblage A, and assemblage B was not found [11, 12], while the aetiological agents of hookworm infections were* A. ceylanicum* and* A. caninum* [13, 14]. Dogs and cats (pet animals) are often infected with these three zoonotic parasites, thus posing great potential risk to public health. Accurate and rapid diagnosis is essential for the formulation of effective control measures.

The benefits of conventional microscopy are mainly due to technical simplicity and low cost. However, neither the species of hookworms nor the assemblage of* G. lamblia* could be identified through the microscopy of eggs or cysts. PCR-based methods have key implication to address this question, and several techniques have been developed for the identification and detection of hookworms or* G. lamblia* at molecular level, such as PCR-restriction fragment length polymorphism (RFLP) [15], single-strand conformation polymorphism (SSCP) [16], and real-time PCR [17]. Even though these approaches are very useful and sensitive, the number of steps to detect these pathogens can be time consuming and quite expensive. Furthermore, the higher the step number, the higher the risk of contamination.

Multiplex PCR assay enables the identification and detection of mixed infection in a single reaction that contains multiple primer pairs; it is a convenient, rapid, cost-effective, and high-efficiency method. So far, several multiplex PCR assays have been documented for the identification and detection of various parasite species [18–20]. Prior to the present study, there have been no reports of multiplex PCR assay for simultaneous detection of* A. ceylanicum, A. caninum, *and* G. lamblia* assemblage A. For the first time, we developed here a multiplex PCR assay for the detection of these three zoonotic parasites, providing a convenient, rapid, and efficient technique for the clinical detection and epidemiological investigation.

## 2. Materials and Methods

### 2.1. Samples

*G. lamblia* WB trophozoites were obtained from professor Zhao-Rong Lun (Center for Parasitic Organisms, State Key Laboratory of Biocontrol, School of Life Sciences, Sun Yat-Sen University, Guangzhou, China); adult hookworms were collected from dog carcasses supplied by veterinary hospital of South China Agricultural University. After cleaning with saline solution,* A. ceylanicum *and* A. caninum* were morphologically identified to species level according to existing keys and descriptions [21], fixed in 70% (v/v) ethanol, and stored at −20°C until use. Control DNA sample of* A. braziliense *was kindly provided by Stephen E. Bienhoff (Novartis Animal Health, USA), control DNA samples of* Toxocara cati*,* Trichuris vulpis*,* Dipylidium caninum*,* Isospora felis, *and* Giardia lamblia *Assemblages C, D, and F were kept in our laboratory.

### 2.2. Genomic DNA Extraction

The extraction of genomic DNA from* A. ceylanicum*,* A. caninum,* and* G. lamblia* WB was performed by using proteinase K treatment, followed by spin-column purification (Wizard SV Genomic DNA Purification System, Promega). For isolation of genomic DNA from fecal samples, the QIAmp DNA stool kit (Qiagen, Hilden, Germany) was used according to the manufacturer's instructions, with the exception that all samples were washed three times with distilled water and then pretreated with 5 cycles of heating at 100°C for 5 minutes and freezing at −80°C for 5 minutes. The extracted DNA was stored at −20°C until use.

### 2.3. Specific Primers Design

Based on the polymorphic sites of ITS1-5.8S rRNA-ITS2,* A. ceylanicum* and* A. caninum* specific primers were designed by Primer Premier 5.0 and combined with the previously published specific primers for* G. duodenalis *assemblage A [22] to generate target fragments of different sizes. The three pairs of specific primers shared similar annealing temperatures and were predicted to form no secondary structures or primer-dimers; their sequences and expected sizes of amplicons are listed in Table 1.

### 2.4. Simplex PCR

Specific PCRs were initially tested for each pair of primers. All PCRs were performed in 25 *μ*L volume containing 2 *μ*L DNA sample, 0.2 *μ*L Taq polymerase (TaKaRa, Dalian, China), 2.5 *μ*L 10x Taq buffer (TaKaRa), 2 *μ*L diethyl-nitrophenyl thiophosphate (dNTP, TaKaRa) mixture, 0.4 *μ*L primers (50 mM), and 17.5 *μ*L of distilled water. The thermocycler program consisted of 94°C for 5 min, followed by 35 cycles of 94°C for 30 s, 59°C for 30 s, 72°C for 30 s, and a final extension step at 72°C for 7 min. For each PCR, negative control (H_2_O) was run together. PCR products were analyzed by electrophoresis on 1.5% agarose gels, stained with 0.2 g/mL of ethidium bromide, and visualized on a UV transilluminator. All three PCR conditions were then optimized by changing a range of parameters including annealing temperatures, the concentration of each specific primer set, and Mg^2+^ to determine the optimum range of all parameters.

### 2.5. Multiplex PCR

Based on the optimization results of simplex PCR, multiplex PCR reaction parameters were optimized. Firstly, all three pairs of primers were added to the one PCR reaction mixture in equal volumes (0.8 *μ*L) to test the interaction effect of the primers. Then the concentrations of primers and Mg^2+^ and annealing temperatures were adjusted to get the optimal conditions.

### 2.6. Specificity and Sensitivity Test

The multiplex PCR was evaluated for specificity using DNA samples representing the common intestinal parasites of dogs and cats, such as* Toxocara cati*,* Trichuris vulpis*,* Dipylidium caninum*,* Isospora felis, *and* G. lamblia *(Assemblages C, D, and F). To determine the minimum number of* A. ceylanicum*/*A. caninum* eggs in fecal samples detectable by the established multiplex PCR, serial dilution samples with 50, 40, 30, 20, 10, 5, and 1 egg(s) were pipetted out from purified eggs. The genomic DNA was extracted using the QIAmp DNA stool kit (Qiagen, Hilden, Germany). Then 2 mL DNA samples mixed with 1 mL 10-fold serial dilutions of genomic DNA (72 ng/*μ*L) extracted from* G. lamblia* assemblage A were amplified by the established multiplex PCR.

### 2.7. Applications of the Multiplex PCR Assay

To test the availability of multiplex PCR, 112 fecal samples, collected through an epidemiologic survey of hookworm infections in cats [23], were used in this study. Data collection on cat age, gender, and neutering were provided by humane shelters. Prevalence (%) was calculated as percentage of positive number in number of cats examined. All data were analyzed using SPSS programme for window version 11.5 (SPSS Inc., Chicago, USA). Chi-square was used to investigate the degree of association between variables and considered *P* < 0.05 as level of significance.

## 3. Results

### 3.1. Specificity and Optimization of Simplex PCR

Specificity test of simplex PCR showed that three different-sized amplicons were amplified: 191 bp for* A. ceylanicum*, 303 bp for* G. lamblia* assemblage A, and 427 bp for* A. caninum*. The optimal parameters in the simplex PCR are shown in Table 2.

### 3.2. Development of a Multiplex PCR Assay

**Figure 1:**
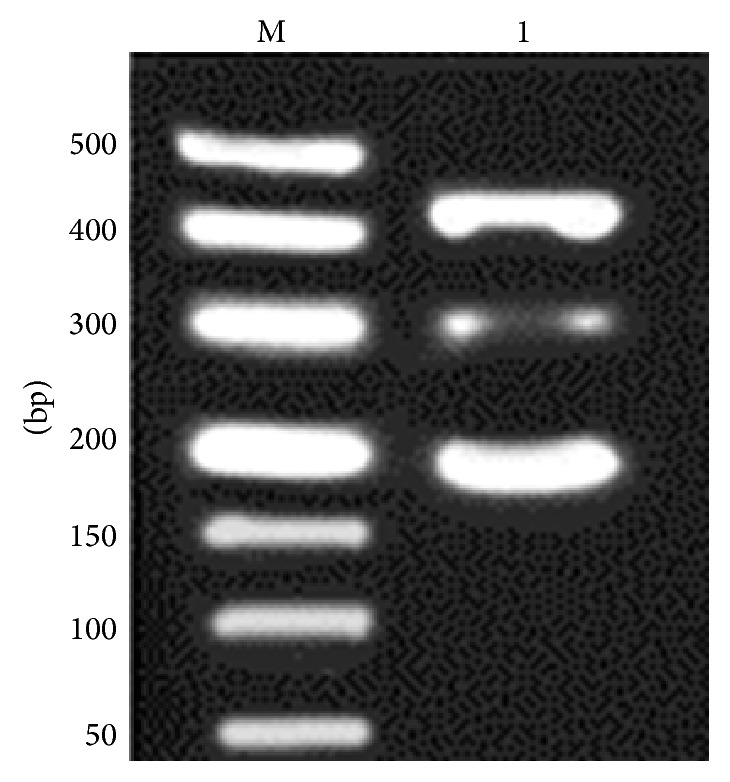
Interaction effect of multiplex PCR primers. M: DL2000 DNA Marker; 1: target fragments amplified simultaneously with three pairs of primers in equal volume.

**Figure 2:**
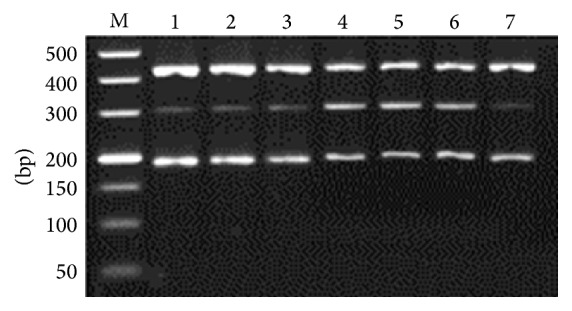
Optimization of* Giardia lamblia* assemblage A primers concentration. M: DL2000 DNA Marker; 1–7: 0.9, 1.0, 1.1, 1.2, 1.3, 1.4, and 1.5 *μ*M, respectively.

**Figure 3 fig3:**
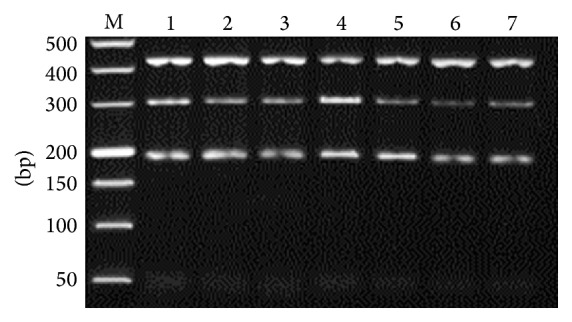
Optimization of annealing temperature. M: DL2000 DNA Marker; 1–7: 53.8, 55.5, 57.2, 58.8, 62.2, 63.7, and 65°C, respectively.

**Figure 4:**
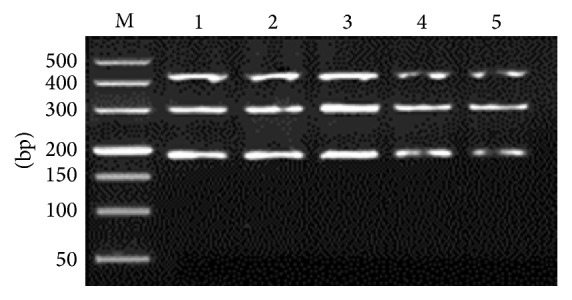
Optimization of Mg^2+^ concentration. M: DL2000 DNA Marker; 1–5: 2.0, 2.5, 3.0, 3.5, and 4.0 *μ*M, respectively.

All three targeted bands were amplified in one tube simultaneously (Figure 1), and* G. lamblia* assemblage A specific primers showed lower amplification efficiency, compared to* A. ceylanicum* and* A. caninum*. Optimal amplification results were obtained with 1.2 *μ*M of* G. lamblia* assemblage A specific primers, at 58.8°C of annealing temperature, and 2.0 mM of MgCl_2_ (Figures 2 [Fig fig3]–4). Then the multiplex PCR was established, where reaction mixture consisted of 2 *μ*L DNA template, 0.2 *μ*L Taq polymerase, 2.5 *μ*L 10x Taq buffer (Mg^2+^ free), 2 *μ*L dNTP mixture, 2 *μ*L MgCl_2_, 0.8 *μ*L primer A.cey-F/R, and A.can-F/R (50 *μ*M) (A.cey-F/R for* A. ceylanicum*; A.can-F/R for* A. caninum*), 1.2 *μ*L primer 5C1-P21-F/R (50 *μ*M) (5C1-P21-F/R for* G. lamblia* assemblage A), and 10.7 *μ*L of distilled water. PCR conditions consisted of an initial denaturation at 94°C for 5 min, followed by 35 cycles of 94°C for 30 s, 58.8°C for 30 s, and 72°C for 35 s. A final elongation was performed at 72°C for 10 min.

### 3.3. Specificity and Sensitivity of Multiplex PCR

**Figure 5:**
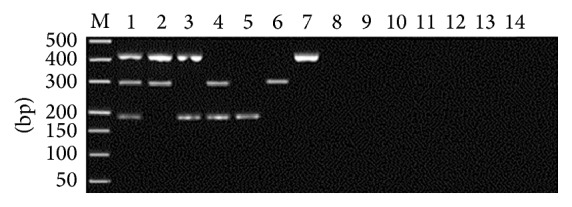
Specificity of multiplex PCR. M: DL2000 DNA Marker; 1:* Ancylostoma ceylanicum*,* A. caninum,* and* G. lamblia* assemblage A; 2:* A. caninum* and* G. lamblia* assemblage A; 3:* A*.* ceylanicum* and* A. caninum*; 4:* A*.* ceylanicum* and* G. lamblia* assemblage A; 5:* A*.* ceylanicum*; 6:* G. lamblia* assemblage A; 7:* A. caninum*; 8:* Toxocara cati*; 9:* Trichuris vulpis*; 10:* A. braziliense*; 11:* Dipylidium caninum*; 12:* Isospora felis*; 13:* G. lamblia* assemblages C, D, and F; 14: Sterile ddH_2_O.

**Figure 6:**
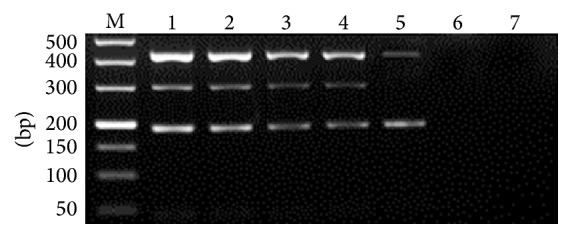
Sensitivity of multiplex PCR. M: DL2000 DNA Marker; 1–7: tenfold serial dilutions of* G. lamblia* assemblage A DNA from 10^0^ to 10^6^, and 50, 40, 30, 20, 10, 5, and 1 egg(s) of* A*.* ceylanicum *or* A. caninum*.

The multiplex PCR was found to specifically amplify the genomic DNA from* A. ceylanicum*,* A. caninum,* and* G. lamblia* assemblage A (any combination). No amplification was observed when the multiplex PCR was performed on genomic DNA of* Toxocara cati*,* Trichuris vulpis*,* Dipylidium caninum*,* Isospora felis*,* Giardia lamblia *assemblages C, D, and F, and sterile ddH_2_O (Figure 5). The sensitivity test revealed that the detection limit was 10 eggs for* A. ceylanicum* and* A. caninum* in fecal sample, and 72 pg DNA for* G. lamblia* assemblage A (Figure 6).

### 3.4. Validation of Multiplex PCR

Of 112 fecal samples, 17.8% were infected with* A. ceylanicum* and 34.8% were infected with* A. caninum*, while only 2.7% were infected with* G. lamblia* assemblage A. Data analysis showed that the prevalence of* A. caninum *was significantly higher in young cats than in adults, while no statistically significant difference was observed in gender and neutering group. In addition, there were no significant differences in the prevalence of* A. ceylanicum *and* G. lamblia *with respect to age, gender, and neutering groups (Table 3).

## 4. Discussion

It is known that* A. ceylanicum*,* A. caninum,* and* G. lamblia* assemblage A are common parasites that infect dogs and cats throughout the world. In addition to the veterinary importance, those parasites can also cause zoonotic diseases in human. To date, many methods have been developed for the identification and detection of those parasites, such as the high-resolution melting (HRM) assay established by Zhang et al., which could identify the* G. lamblia* assemblages A and B [24]; the PCR-RFLP developed by Liu et al. could effectively distinguish* A. ceylanicum* and* A. caninum* [14]; the PCR-RFLP established by Bertrand et al. was an effective tool to detect* G. lamblia* assemblages A and B [25]. However, prior to the present study, there had been no report on the utilization of multiplex PCR approaches for detection of hookworm and* G. lamblia*.

This work describes the development of a multiplex PCR method for simultaneous detection of* A. ceylanicum*,* A. caninum,* and* G. lamblia* assemblage A. In the process of establishing multiple PCR detection methods, the primer design is a key point. Firstly, the selected primer design region must be highly specific to avoid competitive amplification among target fragments. In this study, highly variable ITS sequence of hookworms and TPI gene of* G. lamblia* were chosen, and results showed that three pairs of primers could specifically amplify the target fragments. Secondly, the size of amplified fragments was between 191 bp and 427 bp, and the difference between any two fragments surpassed 100 bp, not only reducing the amplification efficiency imbalance problem [26], but also making the bands easy to distinguish on the electrophoretogram. In addition, annealing temperature of those primers was relatively close, making successful amplification of all fragments under the same temperature.

Regarding specificity, this method was specific to amplify the three target fragments, and no cross-reaction with the common intestinal parasites, such as* Toxocara cati*,* Trichuris vulpis*,* Dipylidium caninum*,* Isospora felis*,* and Giardia lamblia *assemblages C, D, and F, of dogs and cats was observed. As for sensitivity test of* A. ceylanicum* or* A. caninum*, the number of eggs detectable was chosen as criterion of evaluation. Unfortunately, the small size of* G. lamblia* hampered its spiking with fecal sample precisely. Our results showed that the detection limit was 10 eggs for* A. ceylanicum* or* A. caninum* in fecal samples, 72 pg genomic DNA for* G. lamblia* assemblage A, thus implying the high specificity and sensitivity of this assay.

By using the established multiplex PCR detection method in present study, 34.8% of fecal samples were discovered to be positive for* A. caninum*, which is higher than the positive rate of* A. ceylanicum* (17.8%). This is in agreement with the previous results detected by the PCR-RFLP method [23]. Thus it supports the correctness of our previous study, or vice versa, as well as the suggestion that the predominant species of hookworms in cats was* A. caninum* in China, and that the prevalence of this species of hookworm in different susceptible hosts may depend on its geographical distribution [13]. In addition, 2.7% of fecal samples were positive for* G. lamblia* assemblage A. Even though it was lower compared with the reported value of 8.8% in 2013 [12], it still poses a potential risk to public health.

In conclusion, this work presents the first report on the development of a multiplex PCR for simultaneous detection of three important zoonotic parasites:* A. ceylanicum*,* A. caninum,* and* G. lamblia* assemblage A. The results indicate that this method was a rapid and effective method for the identification and detection of those zoonotic parasites. The clinical detection results are in line with those previously detected by PCR-RFLP method and show that the cats in Guangzhou harbor all these three zoonotic parasites. Briefly, humans can become infected by these three zoonotic parasites through oral ingestion and percutaneous migration of infective L3 larvae (*A. ceylanicum, A. caninum*) or oral ingestion of cysts (*G. duodenalis*) from the environment. Therefore, control measures are recommended to prevent and reduce the infection risk from cats to humans.

## Figures and Tables

**Table 1:** Primers for the simultaneous detection of *Ancylostoma ceylanicum*, *A. caninum, *and *Giardia lamblia *assemblage A by multiplex PCR.

Target parasite	Primer name	Amplicon size (bp)	Sequences (5′-3′)
*A. ceylanicum*	A.cey-F	191	CCCCGTTACAGCCCTACGAG
	A.cey-R		TCCTGACAGACAAGTGCCGAACT

*G. lamblia* assemblage A	5C1-P21-F	303	ATGCTAGCCGTAGTTAATAAGG
	5C1-P21-R		ACCGGCCTTATCTACCAGC

*A. caninum*	A.can-F	427	AGCATTAGGCTAACGCCCGA
	A.can-R		AACGAGTTTGCTGTCATTCAGTCC

**Table 2:** Optimization results of simplex PCR.

Parasite	Primers concentration (*μ*M)	Mg^2+^ concentration (mM)	Annealing temperature (°C)
*A. ceylanicum*	0.4–1.2	1.0–4.0	51–65
*A. caninum*	0.4–1.2	1.0–4.0	51–65
*G. lamblia* assemblage A	0.2–1.2	1.0–4.0	51–65

**Table 3:** The prevalence (%) of *A. ceylanicum*, *A. caninum, *and *G. lamblia *assemblage A in 112 cat fecal samples from Guangzhou.

Characteristics	*N*	*A. ceylanicum*	*A. caninum*	*G. lamblia *assemblage A
Age (year)				
<1	42	23.8 (*n* = 10)	52.4 (*n* = 22)^*∗*^	7.1 (*n* = 3)
>1	70	14.3 (*n* = 10)	24.3 (*n* = 17)^*∗*^	—
Gender				
Female	73	17.8 (*n* = 13)	41.1 (*n* = 30)	4.11 (*n* = 3)
Male	39	18.0 (*n* = 7)	23.1 (*n* = 9)	—
Neutering				
Neutered	34	14.7 (*n* = 5)	29.4 (*n* = 10)	2.9 (*n* = 1)
Not neutered	78	19.2 (*n* = 15)	37.1 (*n* = 29)	2.6 (*n* = 2)

Total	112	17.8 (*n* = 20)	34.8 (*n* = 39)	2.7 (*n* = 3)

*N*: number of cats examined; ^*∗*^statistically significant difference, *P* < 0.05.
